# Characterization, Identification and Evaluation of Wheat-*Aegilops sharonensis* Chromosome Derivatives

**DOI:** 10.3389/fpls.2021.708551

**Published:** 2021-07-26

**Authors:** Xiaolu Wang, Zhihui Yu, Hongjin Wang, Jianbo Li, Ran Han, Wenjing Xu, Guangrong Li, Jun Guo, Yan Zi, Faji Li, Dungong Cheng, Aifeng Liu, Haosheng Li, Zujun Yang, Jianjun Liu, Cheng Liu

**Affiliations:** ^1^Crop Research Institute, Shandong Academy of Agricultural Sciences, Jinan, China; ^2^Key Laboratory of Wheat Biology and Genetic Improvement in the North Huang and Huai River Valley, Ministry of Agriculture, Jinan, China; ^3^National Engineering Laboratory for Wheat and Maize, Jinan, China; ^4^School of Life Sciences and Technology, University of Electronic Science and Technology of China, Chengdu, China

**Keywords:** *Aegilops sharonensis*, chromosome derivatives, cytogenetic identification, PLUG marker, powdery mildew resistance

## Abstract

*Aegilops sharonensis*, a wild relative of wheat, harbors diverse disease and insect resistance genes, making it a potentially excellent gene source for wheat improvement. In this study, we characterized and evaluated six wheat-*A. sharonensis* derivatives, which included three disomic additions, one disomic substitution + monotelosomic addition and two disomic substitution + disomic additions. A total of 51 PLUG markers were developed and used to allocate the *A. sharonensis* chromosomes in each of the six derivatives to *Triticeae* homoeologous groups. A set of cytogenetic markers specific for *A. sharonensis* chromosomes was established based on FISH using oligonucleotides as probes. Molecular cytogenetic marker analysis confirmed that these lines were a CS-*A*. *sharonensis* 2S^sh^ disomic addition, a 4S^sh^ disomic addition, a 4S^sh^ (4D) substitution + 5S^sh^L monotelosomic addition, a 6S^sh^ disomic addition, a 4S^sh^ (4D) substitution + 6S^sh^ disomic addition and a 4S^sh^ (4D) substitution + 7S^sh^ disomic addition line, respectively. Disease resistance investigations showed that chromosome 7S^sh^ of *A*. *sharonensis* might harbor a new powdery mildew resistance gene, and therefore it has potential for use as resistance source for wheat breeding.

## Introduction

*Aegilops sharonensis* Eig (Sharon goatgrass, S^sh^S^sh^, 2*n* = 2*x* = 14), a wild relative of wheat, is endemic to the coastal plains of Israel and southern Lebanon (Slageren, 1994), and its genome is closely related to the B genome of common wheat (Olivera and Steffenson, 2009). *A*. *sharonensis* is a diverse source of genes for disease and insect resistance (Gill et al., 1985; Olivera et al., 2007). It has been reported that *A. sharonensis* carries resistance to leaf rust (Snyman et al., 2004; Olivera et al., 2007), stem rust (Valkoun et al., 1985; Olivera et al., 2007), stripe rust (Anikster et al., 2005; Olivera et al., 2007), powdery mildew (Dhaliwal et al., 1993; Olivera et al., 2007), and greenbug (Gill et al., 1985). Moreover, *A. sharonensis* has high tolerance to salt, drought, aluminum, boron, and nutrient deficiencies (Manyowa, 1989; Waines et al., 1993; Xu et al., 1993; Gorny and Garczynski, 2008). Consequently, *A. sharonensis* is potentially an excellent gene source for wheat improvement.

Miller et al. (1982) succeeded in producing and identifying a wheat-*A. sharonensis* addition line, which was due to the preferential transmission of one chromosome from *A. sharonensis*. Subsequently, this chromosome was identified as a gametocidal chromosome 4S^sh^ by cytological methods such as chromosome observation, C-banding and *in situ* hybridization. They also produced a wheat-*A. sharonensis* 4S^sh^ (4D) substitution line (Miller et al., 1982). Later, Xu et al. (1992) also reported that they had succeeded in producing a wheat-*A*. *sharonensis* 4S^sh^ (4D) substitution line by using a nullisomic backcrossing procedure. Millet (2007) developed a tetraploid wheat-*A. sharonensis* amphiploid (genome AABBS^1^S^1^). Yu et al. (2017) identified two novel wheat stem rust resistance genes in *A. sharonensis*. Antonyuk et al. (2009) studied 26 wheat-*A. sharonensis* introgression lines. Recently, both Zhao et al. (2014) and Jiang et al. (2014) developed tetraploid wheat-*A. sharonensis* amphidiploids. However, there are very few reports on the isolation of wheat plants carrying individual *A. sharonensis* chromosomes. Li X. Y. et al. (2019); Li et al. (2020) reported 24 HMW-GSs homozygous lines derived from progenies of cross wheat/*A. sharonensis*, and produced three 1S^sh^ (1A) substitution lines, two 1S^sh^ (1B) substitution lines, three 1S^sh^ (1D) substitution lines and two 1S^sh^ (5D) substitution lines. Therefore, the set of wheat-*A. sharonensis* chromosome lines is still not complete, which greatly limits the mapping and utilization of excellent genes derived from this species in wheat.

In this study, six wheat-*A. sharonensis* chromosome derivatives, including three disomic addition lines, one disomic substitution + monotelosomic addition line, and two disomic substitution + disomic addition lines, were identified by (Polymerase Chain Reaction) PCR-based landmark unique gene (PLUG) markers and fluorescence *in situ* hybridization (FISH) analysis. In addition, the infection types (ITs) of disease resistance, spike and grain characteristics of these wheat-*A. sharonensis* chromosome lines were also investigated to provide useful information for the possible subsequent development of wheat-*A. sharonensis* translocations for wheat genetic improvement.

## Materials and Methods

### Plant Materials

*Triticum aestivum* cv. Jinan17 (JN17) and Jimai22 (JM22) were maintained at the Crop Research Institute, Shandong Academy of Agricultural Sciences in Jinan. *T*. *aestivum* cv. Chinese Spring (CS) was provided by Prof. Z. J. Yang, School of Life Science and Technology, University of Electronic Science and Technology of China, Chengdu. The diploid *A*. *sharonensis* accession (TA1995) was provided by Mr. J. Raupp, Wheat Genetic and Genomic Resources Center, Kansas State University, United States. The CS*-A. sharonensis* amphiploid (JIC-31) and six unidentified CS-*A*. *sharonensis* chromosome lines (JIC-32, JIC-33, JIC-34, JIC-35, JIC-36, and JIC-37) were kindly provided by Prof. S. M. Reader, John Innes Centre, United Kingdom.

### Fluorescence *in situ* Hybridization Analysis

Root tip treatments, chromosome slide preparations, and chromosome counting were according to Liu et al. (2011). Fifteen seeds of each of the materials were germinated for collection of root tips for FISH analysis and fifteen cells of each of the materials were studied. Probes Oligo-pTa535-1, Oligo-pSc119.2-1, and Oligo-(GAA)_8_ were synthesized by Chengdu Ruixin Biological Technology Co., Ltd. Probe sequences, the fluorochromes for probe labeling, FISH protocols and labeled DNA signal detection methods were according to Danilova et al. (2012) and Tang et al. (2014) after comparison with the CS standard FISH map. FISH using Oligo-(GAA)_8_ as a probe could be used to identify wheat chromosomes except 1A, 3D, 4D, 5D, and 6D, as described by Danilova et al. (2012). FISH using Oligo-pSc119.2-1 and Oligo-pTa535-1 probes could identify all 42 wheat chromosomes simultaneously as described by Tang et al. (2014). pTa71 (45S rDNA) contains a 9-kb *Eco*RI fragment isolated from bread wheat (Gerlach and Bedbrook, 1979), which could be used to identify homoeologous groups 1 and 6 of *Triticum* and *Aegilops*. Photomicrographs of FISH chromosomes were taken using an Olympus BX-51 microscope.

### DNA Isolation and PLUG-PCR

Total genomic DNA isolation was according to the protocol of Liu et al. (2006). A total of 526 PLUG primer pairs were synthesized according to Ishikawa et al. (2009). All primer pairs were synthesized by Chengdu Ruixin Biological Technology Co., Ltd., and PCR protocol was according to Ishikawa et al. (2009). In order to obtain high levels of polymorphism, the PCR products were digested with the four-base cutter enzymes *Hae*III or *Taq*I according to Ishikawa et al. (2009) and were separated on 2% agarose gels.

### Disease Resistance Testing

The resistance reactions to stripe rust, leaf rust, stem rust, and powdery mildew of the six suspected CS-*A. sharonensis* derivatives were tested. We investigated the disease resistance data for two consecutive years in 2015 and 2016, and 20 individual plants of each line were investigated each year. CS is highly susceptible to all four pathogens, hence the disease response scoring did not begin until CS was fully infected. According to Wang et al. (2014), the disease responses were scored on a 0–4 rating scale, 0 means immune, 0; indicates nearly immune but showing a small fleck on the leaf, 1 means highly resistant, 2 indicates moderately resistant, 3 means moderately susceptible, and 4 indicates highly susceptible. Scores of 0–2 were classified as resistant and 3–4 as susceptible. The pathogenic race selection and disease response rating scale of the four diseases were all according to Gong et al. (2017). The pathogen inoculation methods for stripe rust, leaf rust and powdery mildew were according to Liu et al. (2013), while stem rust inoculation was according to Han et al. (2018). Stripe rust resistance was determined on adult plants using mixed isolates of races CY32, CY33, and Su-4 in the experimental farmland of School of Life Science and Technology, University of Electronic Science and Technology of China, Chengdu, Sichuan Province. Stem rust resistance was determined on seedlings using mixed isolates of pathotypes 34MKGQM and 21C3CTHSM in the greenhouse of College of Plant Protection, Shenyang Agricultural University, Shenyang, Liaoning Province. Leaf rust resistance was determined on seedlings using mixed isolates of THTT, PHTT, THKS, THTS, and THKT (these isolates are prevalent and highly damaging on wheat crops throughout China) in the greenhouse of College of Plant Protection, Hebei Agricultural University, Baoding, Hebei Province. Powdery mildew resistance was determined on seedlings (in the greenhouse) and also on adult plants (in the field) following inoculation with mixed powdery mildew races collected from four different cities including Jinan, Linyi, Dezhou, and Heze of Shandong Province.

### Spike and Grain Characteristics

Chinese Spring and the six suspected CS-*A. sharonensis* chromosome lines were planted in the field at Jinan in Shandong Province on October 25, 2015 and harvested on June 5, 2016. The spikes were collected for photographs on May 10, 2016 then threshed and the grain extracted when fully mature. The spike and grain characters of these materials were investigated and described according to Li et al. (2006).

## Results

### Cytogenetic Identification of Wheat-*A. sharonensis* Chromosome Lines

**FIGURE 1:**
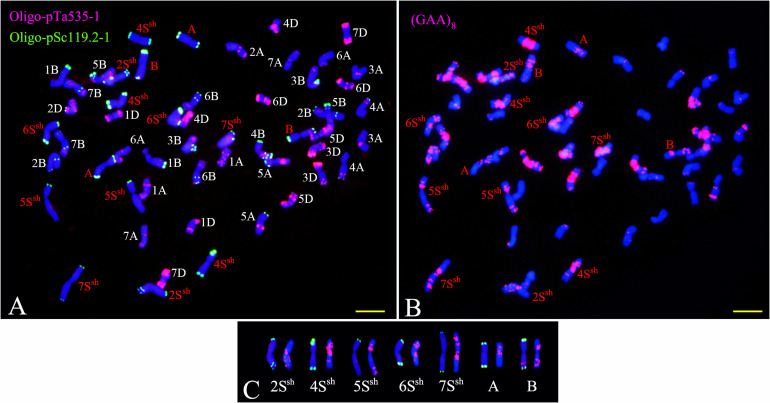
FISH identification of the chromosome constitution of wheat-*Aegilops sharonensis* amphiploid JIC-31. Panel **(A)** shows the probes were Oligo-pSc119.2-1 (green) and Oligo-pTa535-1 (red). Panel **(B)** shows the probe was (GAA)_8_ (red). **(C)** shows the karyotype of the seven pairs of *A. sharonensis* chromosomes in JIC-31; Panel **(A,B)** indicates unidentified chromosome. Bar indicates 10 μm.

**FIGURE 2:**
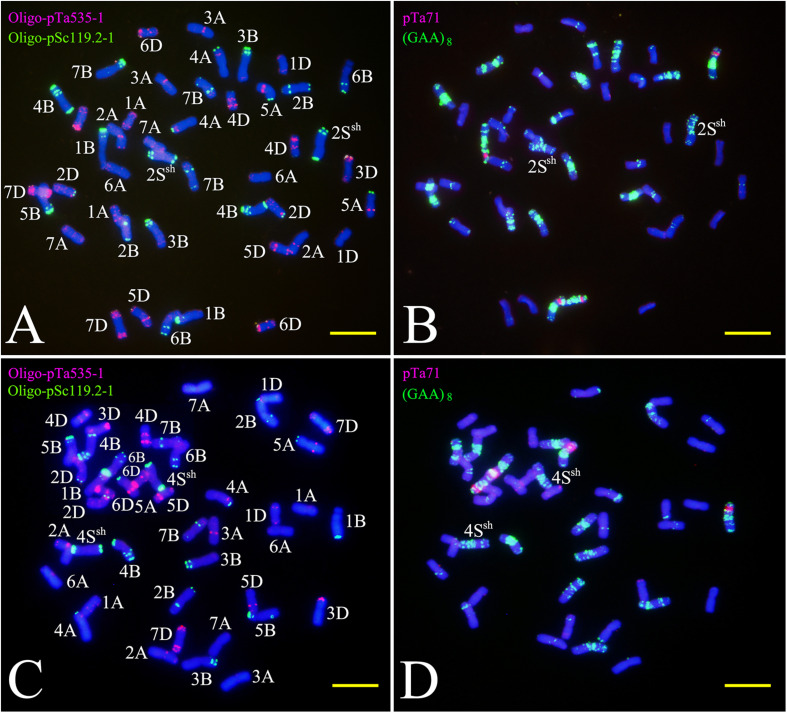
FISH using Oligo-nucleotides as probes on the CS-*A*. *sharonensis* 2S^sh^ disomic addition **(A,B)** and the 4S^sh^ disomic addition **(C,D)**. Panels **(A,C)** are double-color FISH patterns using Oligo-pTa535-1 (red) and Oligo-pSc119.2-1 (green) as probes; **(B,D)** are double-color FISH patterns using pTa71 (red) and (GAA)_8_ (green) as probes. Bar indicates 10 μm.

Sequential FISH with probes Oligo-pSc119.2-1, Oligo-pTa535-1, and Oligo-(GAA)_8_ was used to detect the chromosome constitution of wheat-*A. sharonensis* amphiploid JIC-31 (Figure 1). The karyotype of the seven pairs of *A. sharonensis* chromosomes in JIC-31 is shown in Figure 1C. Cytological studies revealed that the chromosome numbers of JIC-32 to JIC-37 were 44, 44, 42 + monotelosomic, 42, 44, and 44, respectively. FISH on mitotic metaphase chromosomes of these lines showed that the lines JIC-32 and JIC-33 had 42 wheat chromosomes, while the 4D chromosomes in JIC-34 to JIC-37 were missing. A pair of *A*. *sharonensis* chromosomes with distinct FISH signals different from wheat chromosomes was detected in JIC-32, JIC-33, and JIC-35. Two different pairs of *A*. *sharonensis* chromosomes were found in JIC-36 and JIC-37, while disomic and monotelosomic additions of *A*. *sharonensis* chromosomes were detected in JIC-34. Therefore, JIC-32, JIC-33, and JIC-35 are CS-*A*. *sharonensis* disomic addition lines, JIC-36 and JIC-37 are CS-*A*. *sharonensis* disomic substitution + disomic addition lines. JIC-34 is CS-*A. sharonensis* disomic substitution + monotelosomic addition line. FISH patterns of JIC-32 and JIC-33 are shown in Figure 2 (FISH patterns of JIC-34 to JIC-37 are shown in Supplement Figure 1).

**FIGURE 3:**
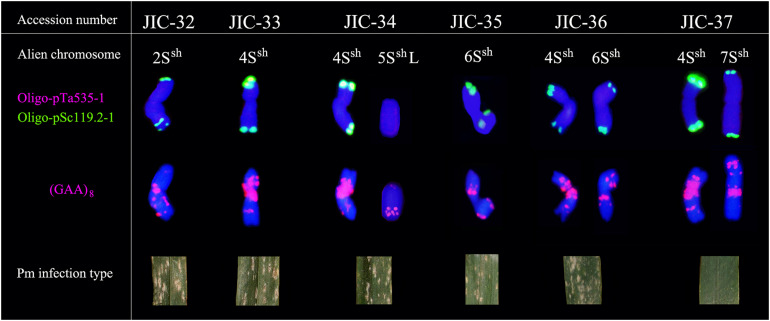
Standard FISH pattern of *A*. *sharonensis* chromosomes using Oligo-nucleotides as probes. Row one indicates alien chromosome; Row two indicates double-color FISH patterns using Oligo-pTa535-1 (red) and Oligo-pSc119.2-1 (green) as probes; Row three indicates FISH patterns using (GAA)_8_ (red) as probes after washing off the double-color FISH signals; Row four indicates photos of powdery mildew in wheat-*A. sharonensis* chromosome lines.

FISH using Oligo-pTa535-1 onto *A. sharonensis* chromosomes in lines JIC-32 to JIC-37 showed no signals associated with that probe. However, slightly different Oligo-pSc119.2-1 signals were found on both terminal regions of all *A. sharonensis* chromosomes (Figure 3). In addition to signals on terminal regions of chromosomes, hybridization associated with Oligo-pSc119.2-1 was observed on sub-terminal regions on the *A. sharonensis* chromosomes in line JIC-32 (Figure 3). Moreover, probe Oligo-(GAA)_8_ with different signal positions and signal strengths, together with chromosome arm ratios, could clearly characterize all *A. sharonensis* chromosomes in lines JIC-32 to JIC-37. The pTa71 signals on the sub-terminal regions could be detected on the short arms of *A. sharonensis* chromosomes in JIC-35 and JIC-36 (Figure not shown), indicating that this pair of *A. sharonensis* chromosomes might be 1S^sh^ or 6S^sh^.

### Molecular Identification of Wheat-*A. sharonensis* Chromosomes

**FIGURE 4:**
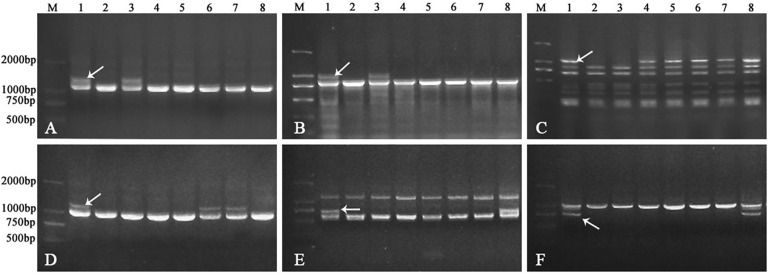
PCR patterns of primer pair TNAC1137 **(A)**, TNAC1197 **(B)**, TNAC1398 **(C)**, TNAC1740 **(D)**, TNAC1867 **(E)**, and TNAC1924 **(F)**. Lane M indicates Marker DM2000. Lanes 1-8 in panels **(A–F)** are CS*-A. sharonensis amphiploid*, CS, CS-*A*. *sharonensis* 2S^sh^ disomic addition, 4S^sh^ disomic addition, 4S^sh^ (4D) substitution + 5S^sh^L monotelosomic addition, 6S^sh^ disomic addition, 4S^sh^ (4D) substitution + 6S^sh^ disomic addition and 4S^sh^ (4D) substitution + 7S^sh^ disomic addition lines, respectively.

In order to identify the homoeologous groups of each of the *A. sharonensis* chromosomes in JIC-32 to JIC-37, a total of 526 PLUG primer pairs were used to develop *A. sharonensis* chromosome-specific markers. As a result, fifty-one primer pairs could generate polymorphisms in *A. sharonensis*, the CS*-A. sharonensis* amphiploid, CS, JM22, and JN17. Among them, four, eight, nine, six, two, five, and seventeen belonged to chromosome homoeologous groups 1–7, respectively (Table 1, 2). The percentage of primers showing polymorphisms across the seven types of *A. sharonensis* chromosomes ranged from 2.6 to 15.6% (Table 1). The PCR patterns of primer pairs TNAC1137, TNAC1197, TNAC1398, TNAC1740, TNAC1867, and TNAC1924 are shown in Figure 4.

**TABLE 1:** PLUG primer pairs screened to identify specific markers of *Aegilops sharonensis* chromosomes.

Chromosome homoeologous groups	Number of PLUG primer pairs	Number of polymorphic markers which could be located on *A. sharonensis* chromosomes	% polymorphism
Group 1	57	4	7.0%
Group 2	67	8	11.9%
Group 3	85	9	10.6%
Group 4	71	6	8.5%
Group 5	78	2	2.6%
Group 6	59	5	8.5%
Group 7	109	17	15.6%
Total	526	51	9.7%

**TABLE 2:** Markers specific for *A. sharonensis* chromosomes developed by the current study.

No.	Primer	Primer sequence (5′–3′)	Wheat chromosomal location	Location on S^sh^ chromosome	Enzyme used	Product size (bp)
1	TNAC1042	F:GACAACAACCCGAACATGC R:ATAGACCCTGATCGGTGCAA	1AL 1BL 1DL	7S^sh^	–	750
2	TNAC1041	F:TCACCACCTCTTTCAGTTGCT R:GCATCAAGGATGAGGAGTCTG	1AL4-0.56-0.61 1BL2-0.69-0.85 1DL9-0.64-1.00	–	*Taq*I	450
3	TNAC1079	F:CACTGTGAAGACCATGATTGC R:TCATCAGGTGGATCAACTTCC	1AL 1BL 1DL	–	*Taq*I	750
4	TNAC1089	F:CGTATGGGAAGATCACAGACC R:TGGTTTCGCATACACATCAAA	1AL 1BL 1DL	–	*Taq*I	350
5	TNAC1102	F:GGAGAGGTGAAGGACCAACTC R:CCTTGCAGCGTAGTGAGATTT	2AS5-0.78-1.00 2BS3-0.84-1.00 2DS5-0.47-1.00	2S^sh^	–/*Taq*I	1,200/1,000
6	TNAC1137	F:GCTGAATCACTCAACCATTCC R:TGCTCGCGCTCTACTTCAC	2AL4-0.27-0.77 2BL4-0.65-0.89 2DL9-0.76-0.94	2S^sh^	–	1,400
7	TNAC1140	F:TCCCAGAAATTACAAGGCTCA R:AGGAACCCTATGCATTGGAAA	2AL3-0.77-1.00 2BL6-0.89-1.00 2DL6-0.94-1.00	2S^sh^	–	700
8	TNAC1142	F:GCCTACGAGTACATGGTCGAG R:CAGCATCCATAACCAGGATGT	2AL3-0.77-1.00 2BL6-0.89-1.00 2DL6-0.94-1.00	2S^sh^	–	1,400
9	TNAC1197	F:CACGGATGACTCTCTCCACAC R:TGGCGACTTGAAGATTTATGC	2AL 2BL 2DL	2S^sh^	–	980
10	TNAC1204	F:GAGAGGAATGCGTGAAGTTTG R:AGACCATCTTTCCGGTCTTTG	2AL4-0.27-0.77 2BL7-0.50-0.58 2DL10-0.49-0.58	2S^*sh*^	–/*Taq*I	800/700
11	TNAC1206	F:ACCTCTACACCAGAGCAGTCG R:CCGAACACCTTGGACACC	2AL 2BL 2DL	2S^sh^	–	950
12	TNAC1176	F:CTTCATGGTTGCTCACGAACT R:CATGCGAAATTTGCTATCCTT	C-2AS5-0.78 2BS11-0.27-0.53 2DS1-0.33-0.41	2S^*sh*^	*Taq*I	1,000
13	TNAC1248	F:ATGATGCAGCAGCAAATTACA R:CTGAGGAGCCTCTCCAACTCT	3AS4-0.45-1.00 C-3BS1-0.33 3DS3-0.24-0.31	–	–	800
14	TNAC1294	F:CGGAAACTTTAGCCTTCTGCT R:GTCGTGTCAGATGCTTTGGAT	3AS4-0.45-1.00 3BS9-0.57-0.78 3DS4-0.59-1.00	–	–	600
15	TNAC1254	F:ATTGATTTCAGCCCTGGAGTT R:CTACTGCACGCACCAGAAGTT	3AL 3BL 3DL	–	*Taq*I	850
16	TNAC1269	F:AACGGTTTGTGTCCTTCAAGA R:CTGAGAAGGACCTGAACAAGC	3AL 3BL 3DL	–	*Taq*I	850
17	TNAC1335	F:CCTATCCAGGTCCGATGCTAT R:GGAAGTTTCTCAAATGCAGGA	C-3AL2-0.21 C-3BL2-0.22 C-3DL1-0.23	–	–	900
18	TNAC1337	F:CTCCTCATCATGCTTCCTCAA R:TCCCTCTCCCAGCTATACTCC	3AL 3BL 3DL	–	–	900, 1,000
19	TNAC1341	F:GTTGAAGCCTACATGCCACAC R:TAGCATGGGCTCCTAACATTG	3AL1-0.26-0.42 C-3BL2-0.22 C-3DL1-0.23	–	*Taq*I	500
20	TNAC1356	F:CGGCAAGTACTCCTTAACACG R:GACGGTCGCGTACAACAAG	3AL3-0.42-0.61 3BL10-0.50-0.63 3DL1-0.23-0.81	–	*Taq*I	350
21	TNAC1365	F:CTTCGGCAGCGATTTCCTA R:GTGAACGTGAGGCCTACTCTG	3AL 3BL 3DL	–	*Taq*I	850
22	TNAC1412	F:CTATGTCCGCAGCCATGAGTA	4AS3-0.76-1.00	4S^sh^	–	1,600
		R:CTTCACACCATCCAAGCTTTC	4BL1-0.71-0.86 4DL11-0.61-0.71			
23	TNAC1416	F:CGGTTTCTGCTTTCATTACCA R:GAGTTGCAGCATTAGCTGGAT	4AS 4BL 4DL	4S^sh^	–	1,800
24	TNAC1396	F:TACCGCTTCCGCTTCTTC R:TGAAATGGAAAGGGAATGTCA	4AL 4BL 4DL	4S^sh^	*Taq*I	1,200
25	TNAC1398	F:CAAGGCAGGTGCTGATATTGT R:ACCCAGGGTTGACTGACATAA	4AS3-0.76-1.00 4BL5-0.86-0.95 4DL12-0.71-0.86	4S^sh^	*Taq*I	1,100
26	TNAC1457	F:TTTGATTCCGTACTGCCTGAG R:GCACCATTTGTTCCAGTCAAC	4AL12-0.43-0.66 4BS1-0.84-1.00 4DS2-0.82-1.00	4S^sh^	*Taq*I	650
27	TNAC1473	F:GAAGCAGCCAATTATTTGTGG R:TCTAGAGGCTCCTTCACATGC	4AL 4BS 4DS	4S^sh^	*Taq*I	700
28	TNAC1455	F:AGCAAACCTCTCCCACGTATT R:ATTCTAGGCAAGGCACTTGGT	5AL 5BL 5DL	5S^sh^	–	750
29	TNAC1621	F:CCTCTCTGCGATCTTCTTGTC R:GGCAGCTCTTGCTTCATCTAA	5AL 5BL 5DL	5S^sh^	–	1,050
30	TNAC1740	F:CGGAAGTGCTCGATTGTATCT R:GCGGGTTTCTTCTCAACCTT	6AL7-0.88-0.90 6BL5-0.40-0.66 6DL6-0.29-0.47	6S^sh^	–/*Taq*I	1,200/250
31	TNAC1748	F:TCGTAGAATTGGTCGACGATG R:ATGGATTGGCAAAGAAAGATG	6AL7-0.88-0.90 6BL8-0.66-0.70 6DL1-0.47-0.68	6S^sh^	*Taq*I	750
32	TNAC1751	F:CTTCCTTTGCTTGTGATCCTG R:GCCTGAGGACTTGAAGTGGTA	6AL8-0.90-1.00 6BL1-0.70-1.00 6DL12-0.68-0.74	6S^sh^	*Taq*I	900
33	TNAC1756	F:CTCCATGGACAATTCCTGCTA R:AAGGCCAGTTCCAGATTCAGT	6AL 6BL 6DL	6S^sh^	*Taq*I	750
34	TNAC1763	F:CGATTGGCCGTACAACTTTC R:TTGATGACGTTGAAGGGTCTC	6AL8-0.90-1.00 6BL1-0.70-1.00 6DL10-0.80-1.00	6S^sh^	*Taq*I	1,000
35	TNAC1867	F:GCCTTTCCTTTGGTAGTCTGG R:CGATCCAAATGATCCTGAAGA	C-7AL1-0.39 7BL2-0.38-0.63 7DL1-0.14-0.30	7S^sh^	–	750
36	TNAC1924	F:TAGCTTTGGAACGATGTGTGG R:TGTGGAGCAGTGCTGTTTATG	7AL 7BL 7DL	7S^sh^	–	750
37	TNAC1801	F:CAGCAACTCAGCTTTGGTCAC R:GCAAGCCTGTTTGGCATTT	7AS 7BS 7DS	7S^sh^	*Hae*III	550
38	TNAC1920	F:CTGTGACGCCCTAGAATCTGA R:CAAGTCGACGGTACTCTCTGG	7AS 7BS 7DS	7S^sh^	*Hae*III	1,500
39	TNAC1843	F:TGGAAAGTCAATCCATTCTGG R:GCGACAAGACTATGGCATTTC	7AL 7BL 7DL	7S^sh^	*Taq*I	800
40	TNAC1881	F:GAAGGGCTATGACCAGCTTCT R:GAAGGGCTATGACCAGCTTCT	7AL 7BL 7DS	7S^sh^	*Taq*I	400
41	TNAC1888	F:AGGGATGTGTTGGAGCTGTTA R:CACAGTGACCTTCTGCTCCTT	C-7AL1-0.39 7BL2-0.38-0.63 7DL5-0.30-0.61	7S^sh^	*Taq*I	750
42	TNAC1902	F:AATACCAGGTCCTCCAACTTT R:TGGAATCGCTGAGAAAGAATG	7AL 7BL 7DL	7S^sh^	*Taq*I	1,500
43	TNAC1922	F:CAGAGCAATAAAGTGCACATGG R:AGAACCAGGGATCAAACGACT	7AS 7BS	7S^sh^	*Taq*I	350
			7DS			
44	TNAC1774	F:CAAGTCTTGGGATGACCTTCA R:GTTGATCATCCGCTTCATCTC	7AS 7DS	7S^sh^	–	1,400
45	TNAC1781	F:AACTGGCAATCAGCAGCAC R:CACCACGCTCTCTTTCATCTT	7AS2-0.73-0.83 7BS2-0.27-1.00 7DS4-0.73-1.00	7S^sh^	–	1,700
46	TNAC1827	F:TCCTCATGTCCAGCAAGGA R:TCCAATTCAATCTCCTGTTGC	7AL 7BL 7DL	7S^sh^	–	750
47	TNAC1948	F:TTTGTCTGTTAGGGCATCAGG R:GTGTATGATGCGAATGGAAGG	7AS8-0.45-0.59 7BS1-0.27-0.27 7DS2-0.61-0.73	7S^sh^	–	1,100
48	TNAC1786	F:CCCTTTCCATATTCTTCCACCT R:GGAAAGAGTATCTTCCTCGTTTGA	7AS 7BS 7DS	7S^sh^	*Taq*I	600
49	TNAC1788	F:CTGTGGAGATGAATGCACAAA R:AGAAGTGGGTCCTTTCCATGT	7AS 7BS 7DS	7S^sh^	*Taq*I	900
50	TNAC1806	F:ATTCCTCGTGAATTGCTGGAT R:TCTGCAGTTAGGGACTTGAAA	7AS8-0.45-0.59 7BS2-0.27-1.00 7DS2-0.61-0.73	7S^sh^	*Taq*I	800
51	TNAC1937	F:AGCGGCATGTGGTAATCAATA R:CGGACGATCGAGAACACC	7AS 7DS	7S^sh^	*Taq*I	600

*Information of wheat chromosomal locations is according to Ishikawa et al. (2007).*

TNAC1102 and another seven primer pairs specific to chromosome homoeologous group 2, could amplify polymorphisms in the *A. sharonensis* chromosomes of JIC-32, indicating that the pair of *A. sharonensis* chromosomes in that line was 2S^sh^. The chromosome number of JIC-32 was 44, including the 42 complete wheat chromosomes, therefore, JIC-32 was a CS-*A*. *sharonensis* 2S^sh^ disomic addition (Figures 2A, B). Based on the results of molecular markers and cytological identification, the same analysis method was performed on JIC-33 to JIC-37, indicating that JIC-33 was a CS-*A*. *sharonensis* 4S^sh^ disomic addition (Figures 2C, D), while JIC-34 was a CS-*A. sharonensis* 4S^sh^ (4D) substitution + 5S^sh^L monotelosomic addition (Supplementary Figures 1A, B), JIC-35 was a CS-*A. sharonensis* 6S^sh^ disomic addition (Supplementary Figures 1C, D), JIC-36 was a CS-*A. sharonensis* 4S^sh^ (4D) substitution + 6S^sh^ disomic addition (Supplementary Figures 1E, F) and JIC-37 was a 4S^sh^ (4D) substitution + 7S^sh^ disomic addition line (Supplementary Figures 1G, H).

### Spike and Grain Characters of Wheat-*A. sharonensis* Chromosome Lines

**FIGURE 5:**
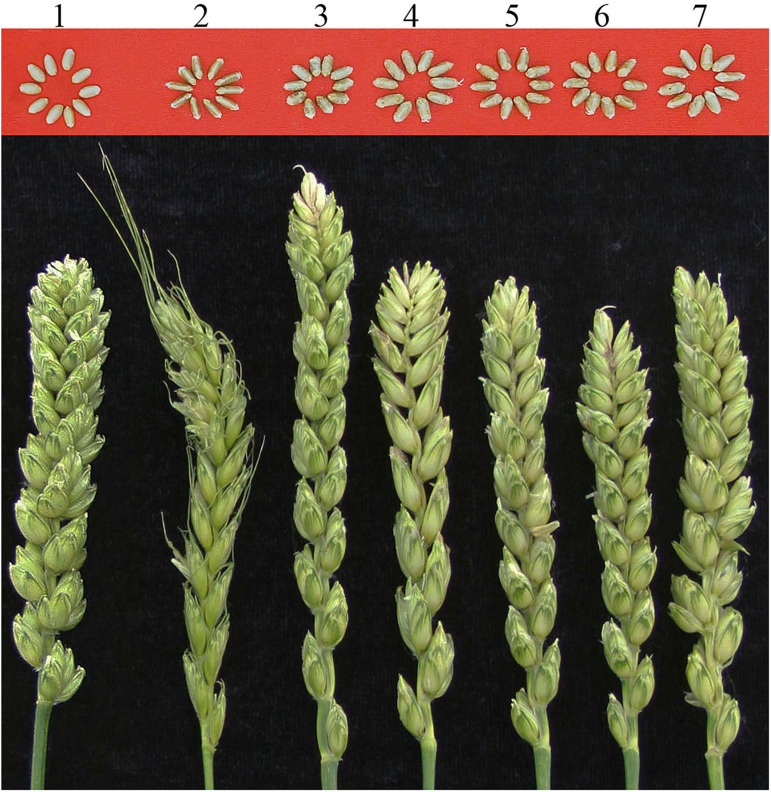
Spike and grain morphologies of wheat-*A. sharonensis* chromosome lines. Grain and spikes from left to right are CS, CS-*A*. *sharonensis* 2S^*sh*^ disomic addition, 4S^*sh*^ disomic addition, 4S^*sh*^ (4D) substitution + 5S^*sh*^L monotelosomic addition, 6S^*sh*^ disomic addition, 4S^*sh*^ (4D) substitution + 6S^*sh*^ disomic addition and 4S^*sh*^ (4D) substitution + 7S^*sh*^ disomic addition lines, respectively.

Compared to spike morphologies of CS, the spikes of the six CS-*A. sharonensis* chromosome derivatives were all varied (Figure 5). Spikes of the CS-*A. sharonensis* 2S^sh^ disomic addition had short awns and narrow spikes. The lower inter-spikelet segments of the heads of CS-*A. sharonensis* 4S^sh^ (4D) substitution + 5S^sh^L monotelosomic addition, 6S^sh^ disomic addition, 4S^sh^ (4D) substitution + 6S^sh^ disomic addition and 4S^sh^ (4D) substitution + 7S^sh^ disomic addition lines were more elongated than that of CS. The CS-*A. sharonensis* 4S^sh^ disomic addition line (JIC-33) showed slightly elongated spikelets and overall longer spikes than that of CS. The CS*-A. sharonensis* 4S^sh^ (4D) substitution + 6S^sh^ disomic addition (JIC-36) showed shorter spikes and fewer spikelets per head than that of CS (Figure 5).

Grain morphologies of the six CS-*A. sharonensis* chromosome derivatives identified above were similar to that of CS, while the CS-*A. sharonensis* 2S^sh^ disomic addition (JIC-32) showed slender grains and darker pericarp color than that of CS (Figure 5) and the 4S^sh^ disomic addition (JIC-33) showed smaller grains than those of CS.

### Disease Resistance Tests of Wheat-*A. sharonensis* Chromosome Lines

Stripe rust, leaf rust, stem rust, and powdery mildew resistance tests showed that all the materials were moderately to highly susceptible to stripe rust, leaf rust, and stem rust (Table 3) except that the infection reaction for leaf rust on the CS-*A. sharonensis* 4S^sh^ (4D) substitution + 7S^sh^ disomic addition (JIC-37) was not obtained. The CS-*A. sharonensis* 4S^sh^ (4D) substitution + 7S^sh^ disomic addition (JIC-37) was nearly immune to powdery mildew, while CS and other CS-*A. sharonensis* chromosome lines tested were highly susceptible to powdery mildew (Table 3), suggesting that chromosome 7S^sh^ of *A. sharonensis* might carry powdery mildew resistant gene(s).

**TABLE 3:** The chromosome composition and Stripe rust, leaf rust, stem rust, and powdery mildew infection types of JIC-32 to JIC-37.

Line	*2n*	Chromosome composition	Stripe rust	Leaf rust	Stem rust	Powdery mildew
JIC-32	44	42W + 2S^sh^2S^sh^	S	S	S	S
JIC-33	44	42W + 4S^sh^4S^sh^	S	S	S	S
JIC-34	42 + monotelosomic	40W + 4S^sh^4S^sh^ + 5S^sh^L5S^sh^L	S	S	S	S
JIC-35	42	40W + 6S^sh^6S^sh^	S	S	S	S
JIC-36	44	40W + 4S^sh^4S^sh^ + 6S^sh^6S^sh^	S	S	S	S
JIC-37	44	40W + 4S^sh^4S^sh^ + 7S^sh^7S^sh^	S	–	S	R

*R, resistant; S, susceptible; −, uninvestigated.*

## Discussion

### Chromosomes Transferred From *A. sharonensis* Into Wheat

Transferring each pair of *A. sharonensis* chromosomes into wheat is difficult due to the presence of gametocidal (*Gc*) genes that control the preferential transmission of chromosome 4S^sh^ (Endo, 1982; Miller et al., 1982). Therefore, it is not easy to produce a complete set of wheat-*A. sharonensis* additions or substitutions (Miller et al., 1982; Olivera and Steffenson, 2009). Maan found a *T. urartu*-*A. sharonensis* amphiploid TA3398 in North Dakota in 1972 (unpublished). King et al. (1991) induced a mutation in the male fertility gene of the preferentially transmitted *A. sharonensis* chromosome 4S^1^ (some scientists defined the genome of *A. sharonensis* as S^1^S^1^). Friebe et al. (2003) produced a mutation of the *A. sharonensis Gc2* gametocidal gene (*Gc2*^*mut*^), which opened a way for introgression of genes from *A. sharonensis* into wheat. Zhang et al. (2001) reported the production of additions 1S^1^, 3S^1^, 5S^1^, 6S^1^, and 7S^1^ in a 4S^1^ (4D) background. Antonyuk et al. (2009) studied 26 wheat-*A. sharonensis* introgression lines which they then separated into six groups based on different substituted chromosomes belonging to definite homoeologous groups and different numbers of translocations. Millet (2007) developed a tetraploid wheat-*A. sharonensis* amphiploid (genome AABBS^1^S^1^). Li X. Y. et al. (2019); Li et al. (2020) reported 24 HMW-GSs homozygous lines derived from progenies of cross wheat/*A. sharonensis*, and produced three 1S^sh^ (1A) substitution lines, two 1S^sh^ (1B) substitution lines, three 1S^sh^ (1D) substitution lines and two 1S^sh^ (5D) substitution lines.

So far, reports regarding the development of wheat-*A. sharonensis* introgression lines are very rare. Furthermore, none to date has reported the production of wheat-*A. sharonensis* 2S^sh^ introgression lines. In this research, six wheat-*A. sharonensis* introgression lines were identified, including a CS-*A*. *sharonensis* 2S^sh^ disomic addition (JIC-32), a 4S^sh^ disomic addition (JIC-33), a 4S^sh^ (4D) substitution + 5S^sh^L monotelosomic addition (JIC-34), a 6S^sh^ disomic addition (JIC-35), a 4S^sh^ (4D) substitution + 6S^sh^ disomic addition (JIC-36) and a 4S^sh^ (4D) substitution + 7S^sh^ disomic addition (JIC-37). Among these six introgression lines, four possessed chromosome 4S^sh^, suggesting that chromosome 4S^sh^ of *A. sharonensis* was transmitted preferentially into wheat due to the gametocidal gene, which confirms the reports of preferential transmission of gametocidal chromosomes of earlier researchers (Endo, 1982; Miller et al., 1982). These six newly identified wheat-*A. sharonensis* chromosome derivatives will enrich the germplasm resources available for wheat breeding.

### Development of New Molecular Markers Specific for *A. sharonensis* Chromosomes

Previous reports regarding identification of useable molecular markers for *A. sharonensis* chromosomes indicated that the percentage which were polymorphic was very low, ranging from 1.3 to 11.4% (Zhang et al., 2001; Zhao et al., 2014; Wei Long, 2016; Li et al., 2020). Zhang et al. (2001) developed 21 RFLP markers to identify CS-*A. sharonensis* 3S^1^, 4S^1^, 5S^1^, 6S^1^, and 7S^1^ addition lines. Antonyuk et al. (2009) used two microsatellite primer pairs to screen wheat-*A. sharonensis* introgression lines. Zhao et al. (2014) used two pairs of primers from 150 SSR markers to identify the S^sh^ genome of *A. sharonensis* among F_1_ hybrids. Wei Long (2016) developed two molecular markers specific to the x- and y-type HMW-GSs genes of *A. sharonensis*, which were validated in accurately tracing and distinguishing *A. sharonensis Glu-1S^sh^* of backcross progenies from *Glu-1A*, *Glu-1B*, and *Glu-1D* of wheat. Li et al. (2020) developed four molecular markers specific to the 1S^sh^ chromosome of *A. sharonensis* from 35 primer pairs.

In this study, we found that 51 PLUG markers from a total of 526 primer pairs could identify the homoeologous groups of each of the *A. sharonensis* chromosomes. Among these primer pairs, four, eight, nine, six, two, five, and seventeen belonged to chromosome homoeologous groups 1–7, respectively (Tables 1, 2). The percentage of each homoeologous group primers generated ranged from 2.6 to 15.6%, with an average percentage of 9.7% (Table 1).

### Powdery Mildew Resistance in *A. sharonensis*

Wild relatives of wheat are an important gene reservoir for resistance to wheat diseases, and have been exploited extensively around the world for wheat improvement (Olivera and Steffenson, 2009). *A. sharonensis*, as well as other wild grasses, has co-evolved in association with many cereal pathogens, such as leaf rust, stem rust, stripe rust, and powdery mildew (Wahl et al., 1984). Among them, the highest frequency and level of resistance reported in *A. sharonensis* was to wheat powdery mildew (Gill et al., 1985; Valkoun et al., 1985; Dhaliwal et al., 1993; Olivera et al., 2007). Zhirov and Ternovskaya (1993) first studied a powdery mildew resistance gene in a wheat-*A. sharonensis* introgression line. Olivera et al. (2008) identified *A. sharonensis* accessions carrying major resistance genes to powdery mildew, and found different genes from accessions native to the southern and northern coastal plains of Israel.

To date, more than 70 powdery mildew resistance genes have been permanently designated (Li G. Q. et al., 2019). Among them, 19 have originated from wheat’s related species (Liu et al., 2019), such as *Pm7*, *Pm8*, *Pm17*, *Pm20*, and *Pm56* from *Secale cereale*, *Pm12*, *Pm32*, and *Pm53* from *A*. *speltoides*, *Pm13* and *Pm66* from *A*. *longissima*, *Pm21* and *Pm55* from *Dasypyrum villosum*, *Pm19*, *Pm34*, *Pm35*, and *Pm58* from *A*. *tauschii*, *Pm29* from *A*. *ovata*, *Pm40* and *Pm43* from *Thinopyrum intermedium*, *Pm51* from *T*. *ponticum* and *Pm57* from *A*. *searsii*. Among the above 19 genes mentioned, none were derived from *A. sharonensis*. In our present study, the CS-*A. sharonensis* 4S^sh^ (4D) substitution + 5S^sh^L monotelosomic addition (JIC-34) and the CS-*A. sharonensis* 4S^sh^ disomic addition line (JIC-33) were highly susceptible to powdery mildew, indicating that there were no powdery mildew resistance genes on chromosomes 4S^sh^ and 5S^sh^L of *A. sharonensis*. However, the CS-*A. sharonensis* 4S^sh^ (4D) substitution + 7S^sh^ disomic addition (JIC-37) was nearly immune to powdery mildew (Table 3), suggesting that the chromosome 7S^sh^ of *A. sharonensis* might carry new powdery mildew resistant gene(s).

## Data Availability Statement

The datasets presented in this study can be found in online repositories. The names of the repository/repositories and accession number(s) can be found in the article/Supplementary Material.

## Author Contributions

CL conceived and designed the experiments. XW, ZHY, HW, JBL, and RH performed the experiments. WX, GL, and JG performed disease resistance testing. YZ, FL, DC, and AL analyzed the data. XW wrote the manuscript. HL, ZJY, JJL, and CL revised the manuscript. All authors contributed to the article and approved the submitted version.

## Conflict of Interest

The authors declare that the research was conducted in the absence of any commercial or financial relationships that could be construed as a potential conflict of interest.

## Publisher’s Note

All claims expressed in this article are solely those of the authors and do not necessarily represent those of their affiliated organizations, or those of the publisher, the editors and the reviewers. Any product that may be evaluated in this article, or claim that may be made by its manufacturer, is not guaranteed or endorsed by the publisher.

## Supplementary Material

Supplementary Figure 1 FISH using Oligo-nucleotides as probes on the CS- *Aegilops sharonensis* 4S^sh^ (4D) substitution + 5S^sh^L monotelosomic addition **(A,B)**, 6S^sh^ disomic addition **(C,D)**, 4S^sh^ (4D) substitution + 6S^sh^ disomic addition **(E,F)**, and 4S^sh^ (4D) substitution + 7S^sh^ disomic addition line **(G,H)**. Panels **(A,C,E,G)** are double-color FISH patterns using Oligo-pTa535-1 (red) and Oligo-pSc119.2-1 (green) as probes; **(B,D,F,H)** are FISH patterns using (GAA)_8_ as probes. Bar indicates 10 μm.

## References

[B1] Anikster Y. Manisterski J. Long D. L. Leonard K. J. (2005). Resistance to leaf rust, stripe rust, and stem rust in *Aegilops* spp. In Israel. *Plant Dis.* 89 303–308. 10.1094/pd-89-0303 30795354

[B2] Antonyuk M. Z. Bodylyova M. V. Ternovskaya T. K. (2009). Genome structure of introgressive lines *Triticum aestivum*/*Aegilops sharonensis*. *Cytol. Genet.* 43 58–67. 20458978

[B3] Danilova T. V. Friebe B. Gill B. S. (2012). Single-copy gene fluorescence *in situ* hybridization and genome analysis: *Acc-2* loci mark evolutionary chromosomal rearrangements in wheat. *Chromosoma* 121 597–611. 10.1007/s00412-012-0384-7 23052335

[B4] Dhaliwal H. S. Singh H. Gill K. S. Randhawa H. S. (1993). “Evaluation and cataloguing of wheat germplasm for disease resistanve and quality,” in *Biodiversity and Wheat Improvement*, ed. Damania A. B. (Chichester, UK: John Wiley and Sons), 123–140.

[B5] Endo T. R. (1982). Gametocidal chromosome of three *Aegilops* species in common wheat. *Canadian J. Genet. Cytol.* 24 201–206. 10.1139/g82-020 33356898

[B6] Friebe B. Zhang P. Gill B. S. Nasuda S. (2003). Characterization of a knock-out mutation at the *Gc2* locus in wheat. *Chromosoma* 111 509–517. 10.1007/s00412-003-0234-8 12684822

[B7] Gerlach W. L. Bedbrook J. R. (1979). Cloning and characterization of ribosomal RNA genes from wheat and barley. *Nucleotide Acids Res.* 7 1869–1885. 10.1093/nar/7.7.1869 537913 PMC342353

[B8] Gill B. S. Sharma H. C. Raupp W. J. Browder L. E. Hatchett J. H. Harvey T. L. (1985). Evaluation of *Aegilops* species for resistance to wheat powdery mildew, wheat leaf rust, hessian fly and greenbug. *Plant Dis.* 69 314–316.

[B9] Gong W. P. Han R. Li H. S. Song J. M. Yan H. F. Li G. Y. (2017). Agronomic traits and molecular marker identification of wheat–*Aegilops caudata* addition lines. *Front. Plant Sci.* 8:1743. 10.3389/fpls.2017.01743 29075275 PMC5644244

[B10] Gorny A. G. Garczynski S. (2008). Nitrogen and phosphorus efficiency in wild and cultivated species of wheat. *J. Plant Nutr.* 31 263–279. 10.1080/01904160701853878

[B11] Han R. Li T. Y. Gong W. P. Li H. S. Song J. M. Liu A. F. (2018). New resistance sources of wheat stem rust and molecular markers specific for relative chromosomes that the resistance genes are located on. *Scientia Agric. Sinica* 51 1223–1232.

[B12] Ishikawa G. Nakamura T. Ashida T. Saito M. Nasuda S. Endo T. R. (2009). Localization of anchor loci representing five hundred annotated rice genes to wheat chromosomes using PLUG markers. *Theoretical Appl. Genet.* 118 499–514. 10.1007/s00122-008-0916-y 19057889

[B13] Ishikawa G. Yonemaru J. Saito M. Nakamura T. (2007). PCR-based landmark unique gene (PLUG) markers effectively assign homoeologous wheat genes to A, B and D genomes. *BMC Genomics* 8:135. 10.1186/1471-2164-8-135 17535443 PMC1904201

[B14] Jiang Q. T. Zhao Q. Z. Yang Q. Ma J. Zhang X. W. Wang C. S. (2014). Amphidiploids between tetraploid wheat and *Aegilops sharonensis* Eig exhibit variations in high-molecular weight glutenin subunits. *Genet. Resour. Crop Evol.* 61 299–305. 10.1007/s10722-013-0072-3

[B15] King I. P. Koebner R. M. D. Reader S. M. Miller T. E. (1991). Induction of a mutation in the male fertility gene of the preferentially transmitted *Aegilops sharonensis* chromosome 4S^1^ and its application for hybrid wheat production. *Euphytica* 54 33–39. 10.1007/bf00145628

[B16] Li G. Q. Cowger C. Wang X. W. Carver B. F. Xu X. Y. (2019). Characterization of *Pm65*, a new powdery mildew resistance gene on chromosome 2AL of a facultative wheat cultivar. *Theoretical Appl. Genet.* 132 2625–2632. 10.1007/s00122-019-03377-2 31214740

[B17] Li X. Y. Wang Q. Li S. Y. Ma J. Wang J. R. Qi P. F. (2019). Stable expression and heredity of alien Glu-1Ssh in wheat-*Aegilops sharonensis* hybrid progenies. *Genet. Resour. Crop Evol.* 66 619–632. 10.1007/s10722-018-00736-8

[B18] Li L. H. Li X. Q. Yang X. M. (2006). *Description Specification and Data Standard of Wheat Germplasm Resources.* Beijing: China Agriculture Press.

[B19] Li X. Y. Li Y. Karim H. Li Y. Zhong X. J. Tang H. P. (2020). The production of wheat-*Aegilops sharonensis* 1S^sh^ chromosome substitution lines harboring alien novel high-molecular-weight glutenin subunits. *Genome* 63 155–167. 10.1139/gen-2019-0106 31846356

[B20] Liu C. Gong W. P. Han R. Guo J. Li G. R. Li H. S. (2019). Characterization, identification and evaluation of a set of wheat-*Aegilops comosa* chromosome lines. *Sci. Rep.* 9 4773–4784. 30886203 10.1038/s41598-019-41219-9 PMC6423130

[B21] Liu C. Li G. R. Yang Z. J. Feng J. Zhou J. P. Ren Z. L. (2006). Specific DNA band isolation and SCAR marker construction of rye genome. *Acta Botanica Boreali-Occidentalia Sinica* 26 2434–2438.

[B22] Liu C. Qi L. L. Liu W. X. Zhao W. C. Wilson J. Friebe B. (2011). Development of a set of compensating *Triticum aestivum-Dasypyrum villosum* Robertsonian translocation lines. *Genome* 54 836–844. 10.1139/g11-051 21961939

[B23] Liu C. Yan H. F. Gong W. P. Li G. R. Liu D. Q. Yang Z. J. (2013). Screening of new resistance sources of wheat leaf rust. *J. Plant Genet. Resour.* 14 935–943.

[B24] Manyowa N. M. (1989). *The Genetics of Aluminum, Excess Boron, Copper and Manganese Stress Tolerance in the Tribe Triticeae and its Implications for Wheat Improvement.* Ph.D. thesis. Cambridge: Cambridge University.

[B25] Miller T. E. Hutchinson J. Chapman V. (1982). Investigation of a preferentially transmitted *Aegilops sharonensis* chromosome in wheat. *Theoretical Appl. Genet.* 61 27–33. 10.1007/bf00261506 24271370

[B26] Millet E. (2007). Exploitation of *Aegilops* species of section Sitopsis for wheat improvement. *Israel J. Plant Sci.* 55 277–287. 10.1560/ijps.55.3-4.277

[B27] Olivera P. D. Anikster Y. Kolmer J. A. Steffenson B. J. (2007). Resistance of Sharon goatgrass (*Aegilops sharonensis*) to fungal diseases of wheat. *Plant Dis.* 91 942–950. 10.1094/pdis-91-8-0942 30780426

[B28] Olivera P. D. Millet E. Anikster Y. Steffenson B. J. (2008). Genetics of resistance to wheat leaf rust, stem rust, and powdery mildew in *Aegilops sharonensis*. *Phytopathology* 98 353–358. 10.1094/phyto-98-3-0353 18944087

[B29] Olivera P. D. Steffenson B. J. (2009). *Aegilops sharonensis*: origin, genetics, diversity, and potential for wheat improvement. *Botany* 87 740–756. 10.1139/b09-040 33356898

[B30] Slageren M. W. V. (1994). *Wild Wheats: A Monograph of Aegilops L. and Amblyopyrum (Jaub. & Spach) Eig (Poaceae).* Wageningen: Agricultural University Wageningen, the Netherlands.

[B31] Snyman J. E. Pretorius Z. A. Kloppers F. J. Marais G. F. (2004). Detection of adult-plant resistance to *Puccinia triticina* in a collection of wild *Triticum* species. *Genet. Resour. Crop Evol.* 51 591–597. 10.1023/b:gres.0000024652.49194.ac

[B32] Tang Z. Yang Z. J. Fu S. L. (2014). Oligonucleotides replacing the roles of repetitive sequences pAs1, pSc119.2, pTa-535, pTa71, CCS1, and pAWRC.1 for FISH analysis. *J. Appl. Genet.* 55 313–318. 10.1007/s13353-014-0215-z 24782110

[B33] Valkoun J. Hammer K. Kucerova D. Bartos P. (1985). Disease resistance in the genus *Aegilops* L. -stem rust, leaf rust, stripe rust, and powdery mildew. *Kulturp Flanze* 33 1133–1153.

[B34] Wahl I. Anikster Y. Manisterski J. Segal A. (1984). “Evolution at the center of origin,” in *The Cereal Rusts. Vol. I, Origins, Specificity, Structure, and Physiology*, eds Roelfs A. P. Bushnell W. R. (Orlando, FL: Academic Press), 33–77.

[B35] Waines J. G. Rafi M. M. Ehdaie B. (1993). “Yield components and transpiration efficiency in wild wheats,” in *Biodiversity and Wheat Improvement*, ed. Damania A. B. (Chichester, UK: John Wiley and Sons), 173–186.

[B36] Wang Z. Cui Y. Chen Y. Zhang D. Liang Y. Zhang D. (2014). Comparative genetic mapping and genomic region collinearity analysis of the powdery mildew resistance gene *Pm41*. *Theoretical Appl. Genet.* 127 1741–1751. 10.1007/s00122-014-2336-5 24906815

[B37] Wei Long (2016). *Development of the Wheat Lines Harboring the Alien HMW-GSs from Aegilops Sharonensis.* Master Dissertation. Chengdu: Sichuan Agricultural University.

[B38] Xu X. Li Z. S. Chen S. Y. Fu J. (1992). Studies on the nullisomic backcrossing procedures for producing alien substitution lines of *Triticum-Aegilops*. *Acta Botanica Boreali-Occidentalia Sinica* 12 9–16.

[B39] Xu X. Monneveux P. Damania A. B. Zaharieva M. (1993). Evaluation for salt tolerance in genetic resources of *Triticum* and *Aegilops* species. *Plant Genet. Resour. Newsletter* 96 11–16.

[B40] Yu G. Champouret N. Steuernagel B. Olivera P. D. Simmons J. Williams C. (2017). Discovery and characterization of two new stem rust resistance genes in *Aegilops sharonensis*. *Theoretical Appl. Genet.* 130 1207–1222. 10.1007/s00122-017-2882-8 28275817 PMC5440502

[B41] Zhang H. Reader S. M. Liu X. Jia J. Z. Gale M. D. Devos K. M. (2001). Comparative genetic analysis of the *Aegilops longissima* and *Ae. sharonensis* genomes with common wheat. *Theoretical Appl. Genet.* 103 518–525. 10.1007/s001220100656

[B42] Zhao Q. Z. Jiang Q. T. Yang Q. Ma J. Wang J. R. Chen G. Y. (2014). Characterization of intergeneric hybrid between common wheat and *Aegilops sharonensis* (Eig) and transfer of alien high molecular weight glutenin subunits into wheat. *Cereal Res. Commun.* 42 640–647. 10.1556/crc.2014.0001

[B43] Zhirov E. G. Ternovskaya T. K. (1993). Transfer of the chromosome conferring mildew resistance from *Aegilops sharonensis* Eig into *Triticum aestivum* L. *Genetika* 29 639–645.

