# Supplementary figures and images for: Characterization, Identification and Evaluation of Wheat-Aegilops sharonensis Chromosome Derivatives
Source: Front Plant Sci. 2021 Jul 26;12:708551. doi: 10.3389/fpls.2021.708551 (PMC8350781; doi:10.3389/fpls.2021.708551)

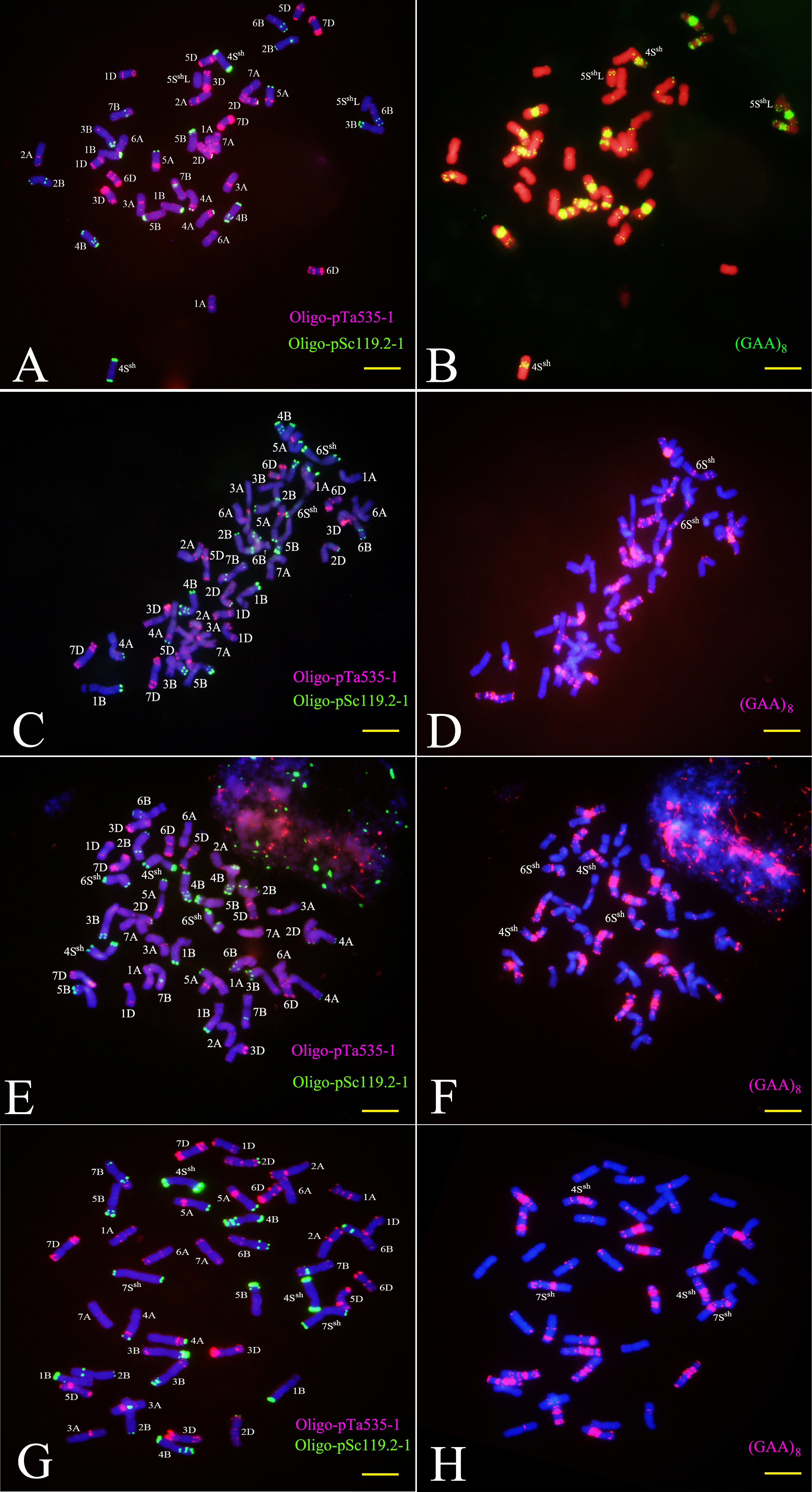

Supplement: Supplementary Figure 1 — FISH using Oligo-nucleotides as probes on the CS- Aegilops sharonensis 4Ssh (4D) substitution + 5SshL monotelosomic addition (A,B), 6Ssh disomic addition (C,D), 4Ssh (4D) substitution + 6Ssh disomic addition (E,F), and 4Ssh (4D) substitution + 7Ssh disomic addition line (G,H). Panels (A,C,E,G) are double-color FISH patterns using Oligo-pTa535-1 (red) and Oligo-pSc119.2-1 (green) as probes; (B,D,F,H) are FISH patterns using (GAA)8 as probes. Bar indicates 10 μm. [file Image_1.JPEG]
